# PS-21 - FACTORS ASSOCIATED WITH VACCINATION UPTAKE IN THE UK AMONG FLUSURVEY 2.0 PARTICIPANTS

**DOI:** 10.1093/pubmed/fdag046.067

**Published:** 2026-07-09

**Authors:** Dr Lauren Adams, Dr Rebecca Green, Dr Gavin Dabrera, Dr Conall Watson

**Affiliations:** UK Health Security Agency; UK Health Security Agency; UK Health Security Agency; UK Health Security Agency

## Abstract

**Background:**

Influenza and COVID-19 vaccination programmes are critical for reducing morbidity and mortality in older adults, yet uptake remains uneven across populations. FluSurvey is a participatory online UK surveillance system that collects self-reported data on symptoms, health behaviours, and vaccination status, providing a valuable platform to investigate determinants of vaccine uptake.

**Objectives:**

This study examines demographic, socioeconomic, and behavioural predictors of vaccination uptake among UK participants in FluSurvey across the 2023–2024 and 2024–2025 seasons and examines reported reasons for and against vaccination among older adults in the UK.

**Methods:**

We analysed FluSurvey data from participants in 2023–2024 and 2024–2025 aged ≥65 years. Descriptive analyses were conducted to examine self-reported reasons for and against vaccination across seasons. Logistic regression was used to examine factors associated with influenza and COVID-19 vaccination uptake. Model 1 adjusted for age and sex, while Model 2 additionally adjusted for IMD and educational attainment.

**Results:**

Vaccination uptake was consistently and strongly associated with prior vaccination behaviour across both seasons, with individuals vaccinated against COVID-19 more likely to receive influenza vaccination, and vice versa. Socioeconomic gradients were evident, with lower uptake among individuals in more deprived IMD quintiles and those with lower educational attainment. Associations observed in age- and sex-adjusted models for employment status, public transport use, and smoking were attenuated after additional adjustment, indicating confounding by socioeconomic and educational factors. Smoking remained negatively associated with influenza vaccination in fully adjusted models, while associations with chronic conditions were weak and inconsistent. Patterns were broadly similar for both influenza and COVID-19 uptake.

**Conclusions:**

Prior vaccination behaviour is the strongest predictor of vaccine uptake in older adults. Many observed associations with behavioural and access-related factors are explained by underlying socioeconomic inequalities. Interventions to improve vaccination coverage should prioritise reducing structural barriers and targeting underserved populations.

